# Distinct nuclear localization signals confer differential microtubule dependence among ZFTA fusion oncoproteins

**DOI:** 10.1016/j.jbc.2026.113320

**Published:** 2026-07-13

**Authors:** Masaki Ishii, Naoki Suto, Ruri Kojima, Kazuaki Katakawa, Kosho Makino, Sachiko Toma-Fukai, Takeshi Tsusaka, Shunsuke Sueki, Masahiro Anada, Hirotatsu Kojima, Shinya Ohata

**Affiliations:** 1 Research Institute of Pharmaceutical Sciences, Faculty of Pharmacy, Musashino University, Nishitokyo, Tokyo, Japan; 2 Drug Discovery Initiative (DDI), Graduate School of Pharmaceutical Sciences, The University of Tokyo, Bunkyo, Tokyo, Japan; 3 Department of Clinical Pharmacy, Faculty of Pharmaceutical Sciences, Shonan University of Medical Sciences, Yokohama, Kanagawa, Japan; 4 Graduate School of Science and Technology, Nara Institute of Science and Technology, Ikoma, Nara, Japan

**Keywords:** colchicine, dynein, ependymoma, microtubule, nuclear localization, ZFTA

## Abstract

Ependymomas are glial tumors that exhibit high resistance to chemotherapy. ZFTA-fusion-positive supratentorial ependymoma (ST-EPN-ZFTA) represents a subclass with a poor prognosis. ST-EPN-ZFTA expresses fusion proteins, which consist of zinc finger translocation-associated (ZFTA) combined with a transcriptional regulator, predominantly RELA. Unlike wild-type RELA, which translocates to the nucleus in a stimulus-dependent manner, ZFTA-RELA constitutively localizes to the nucleus and induces oncogenic gene expression. To identify inhibitors of this aberrant gene expression, 9600 structurally diverse compounds were screened using a ZFTA-RELA-responsive luciferase reporter system. The screen identified a colchicine derivative, and subsequent evaluation revealed colchicine, a microtubule polymerization inhibitor, as the most potent inhibitor (IC_50_ of 90 nM). Colchicine attenuated ZFTA-RELA-upregulated endogenous genes, including *L1CAM*. Colchicine and another microtubule polymerization inhibitor, vinblastine, partially inhibited ZFTA-RELA nuclear localization, similar to the dynein motor protein inhibitors, ciliobrevin D and dynarrestin. This inhibition was mediated through the RELA region of ZFTA-RELA, indicating that RELA retains its microtubule-dependent nuclear import machinery, even in a fusion scenario. Among other ZFTA-fusion proteins, colchicine reduced nuclear localization of ZFTA-NCOA2 and ZFTA-MKL2, but not ZFTA-MAML2 and ZFTA-MAML3. In these fusion proteins, we identified three candidate nuclear localization signals (NLS) exhibiting colchicine-sensitive nuclear localization activity, two within the ZFTA regions absent in ZFTA-RELA and one within MKL2. The results indicate that the microtubule dependence of nuclear localization varies among different ZFTA-fusion partners, which may be attributable to unique NLS sequences in each fusion protein. Our results suggest that microtubule-dependent nuclear transport represents a therapeutic target in ZFTA-fusion-positive tumors.

Ependymomas are primary brain and spinal tumors of the ependymal multiciliated epithelial cells, which line the walls of the ventricles (1, 2, 3, 4, 5). They represent the third most common pediatric brain tumor. These tumors are classified based on their location into three anatomical types: supratentorial, posterior fossa, and spinal (1, 2, 3, 4). Despite advances in molecular classification, which have identified distinct subtypes with varying prognostic implications, ependymomas present significant clinical challenges because of their molecular heterogeneity and aggressive behavior. High recurrence rates and limited treatment options besides surgery and radiotherapy emphasize the need for a deeper understanding of ependymoma biology to develop more effective, molecularly targeted therapies.

Of these anatomical classifications, the molecular characterization of supratentorial ependymomas has revealed distinct subgroups with unique genetic alterations and clinical outcomes (1, 2, 3, 4). A major subclass of supratentorial ependymomas with poor outcome expresses fusion proteins, which consist of a DNA-binding nuclear protein, zinc finger translocation-associated (ZFTA, formerly known as C11orf95), fused with transcriptional regulators containing a transactivation domain, such as *v-rel* reticuloendotheliosis viral oncogene homolog A (RELA), and nuclear receptor coactivator 2 (NCOA2) (6, 7, 8, 9, 10). These ZFTA-fusion-positive supratentorial ependymomas are referred to as ST-EPN-ZFTA. In these fusion proteins, the ZFTA portion recognizes specific DNA sequences, whereas the fusion partners provide a transactivation domain, resulting in ST-EPN-ZFTA through abnormal gene expression (9, 11). Notably, ZFTA also forms fusion proteins with another transcriptional regulator, megakaryoblastic leukemia protein 2 (MKL2), in chondroid lipomas (12, 13). Compounds that inhibit aberrant gene expression by ZFTA-fusion proteins are promising therapeutic candidates for ST-EPN-ZFTA. Therefore, we and others have screened for such compounds (14, 15, 16); however, there are no effective chemotherapeutic regimens for patients with ST-EPN-ZFTA.

ZFTA in ST-EPN-ZFTA most commonly partners with RELA, an effector transcription factor of the nuclear factor-kappa B (NF-κB) pathway (hereafter the fusion protein is referred to as ZFTA-RELA) (6, 7, 8). The NF-κB signaling pathway transduces extracellular stimuli, such as stress and inflammatory cytokines, into intracellular responses and is involved in various cellular processes, including tumorigenesis (17, 18, 19). Unlike wild-type RELA, which translocates to the nucleus in a stimulus-dependent manner, ZFTA-RELA constitutively localizes to the nucleus, even in the absence of stimulation, to cause aberrant transactivation that results in ST-EPN-ZFTA development (6, 7, 8, 9, 20, 21, 22, 23). Targeting ZFTA-fusion proteins is expected to have minimal toxicity to normal tissues, as ZFTA knockout mice develop normally without apparent defects (24). Given that the nuclear levels of ZFTA-RELA are important for tumor growth (25), elucidating the nuclear transport mechanism of ZFTA-RELA may provide insight into the pathogenesis of ST-EPN-ZFTA and lead to the development of ZFTA-targeted therapies.

The nonfused form of RELA interacts with importin α/β through its nuclear localization signal (NLS). This complex is transported to the nucleus along microtubules by dynein motor proteins (26, 27, 28, 29). Because ZFTA-RELA lacks only the initial three residues of RELA and retains all of the functional domains of RELA (6, 7, 8), the microtubule–dynein-dependent nuclear transport machinery of RELA may also be involved in ZFTA-RELA trafficking. However, two important questions remain to be fully addressed: 1) how does ZFTA-RELA translocate to the nucleus, and 2) is there a common mechanism for nuclear transport across different ZFTA-fusion proteins. In addition, no compounds have yet been identified that can inhibit the nuclear migration of these fusion proteins, which may represent therapeutic agents.

In this study, we identified inhibitors of aberrant gene expression induced by ZFTA-RELA and elucidated their mechanism of action. Through a screen of 9600 compounds, we identified a derivative of colchicine as a potent candidate inhibitor. Our results indicate that pharmacological inhibition of microtubule polymerization impairs the nuclear localization of multiple ZFTA-fusion proteins, including ZFTA-RELA, ZFTA-NCOA2, and ZFTA-MKL2. We also identified peptide sequences that exhibit microtubule-dependent nuclear localization activity within different fusion variants. These results indicate that the microtubule dependence of nuclear localization varies among ZFTA-fusion proteins.

## Results

### Colchicine and its derivatives attenuate NF-κB-responsive luciferase activity induced by ZFTA-RELA

**Figure 1:**
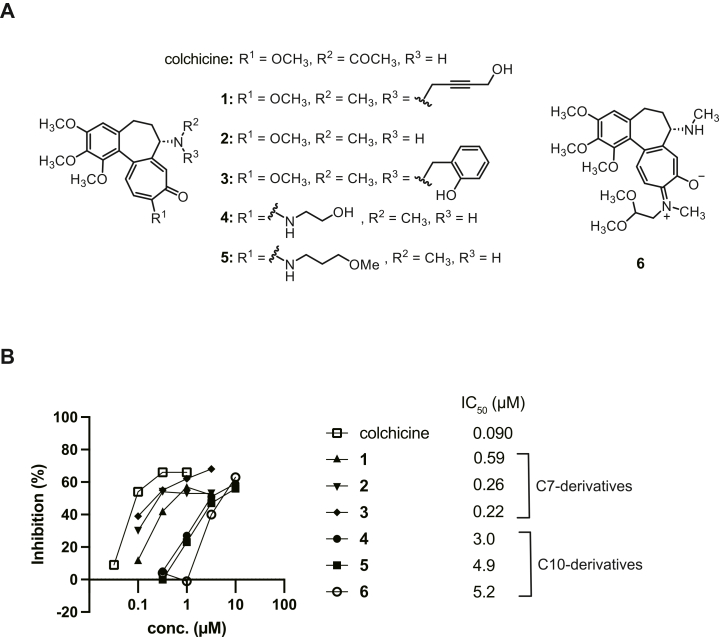
**Comparison of the inhibitory activity of colchicine derivatives on NF-κB-responsive luciferase activity induced by ZFTA-RELA.** (*A*) Structures of colchicine and its derivatives (compounds **1**–**6**). (*B*) Titration assays of colchicine and its derivatives (**1**–**6**) in the inhibition of ZFTA-RELA-induced NF-κB responsive luciferase activities. Data are shown as the mean of four independent experiments, with individual replicate values listed in Table S1.

We previously established the HEK293-derived cell line 6E8, which expresses Flag-tagged ZFTA-RELA (ZFTA-RELA-Flag) and exhibits NF-κB-responsive luciferase activity in a doxycycline concentration-dependent manner (14). Using this cell line and the established screening system (14, 15), we screened the DDI core library, which consists of 9600 chemicals selected for structural diversity, and identified a derivative (Fig. 1*A* (**1**)) of colchicine, a tubulin polymerization inhibitor, as an inhibitor of NF-κB-responsive luciferase activity induced by ZFTA-RELA expression. To identify the most suitable colchicine-related compound for subsequent functional studies, we prepared colchicine and five additional derivatives of colchicine (compounds **2**–**6** in Fig. 1*A*) and compared their inhibitory activity against NF-κB-responsive luciferase using 6E8 cells (Fig. 1*B* and Table S1). Colchicine exhibited the highest potency with an IC_50_ value of 0.090 μM, whereas C7-derivatives, including compounds **1**, **2** (*N*-deacetyl-*N*-methylcolchicine), and **3** (speciosine) also exhibited submicromolar inhibitory activity (IC_50_ 0.22–0.59 μM). In contrast, C10-derivatives in which the C10-methoxy group was substituted with alkylamino or iminium moieties (compounds **4**–**6**) showed reduced inhibitory potency (IC_50_ 3.0–5.2 μM). Based on these results, we selected colchicine for subsequent studies because of its superior inhibitory activity against NF-κB-responsive luciferase activity (lowest IC_50_), its established pharmacological profile, and anti-inflammatory properties, including NF-κB inhibition (26, 27, 30, 31).

### Colchicine attenuation of endogenous ZFTA-RELA-responsive gene expression

**Figure 2:**
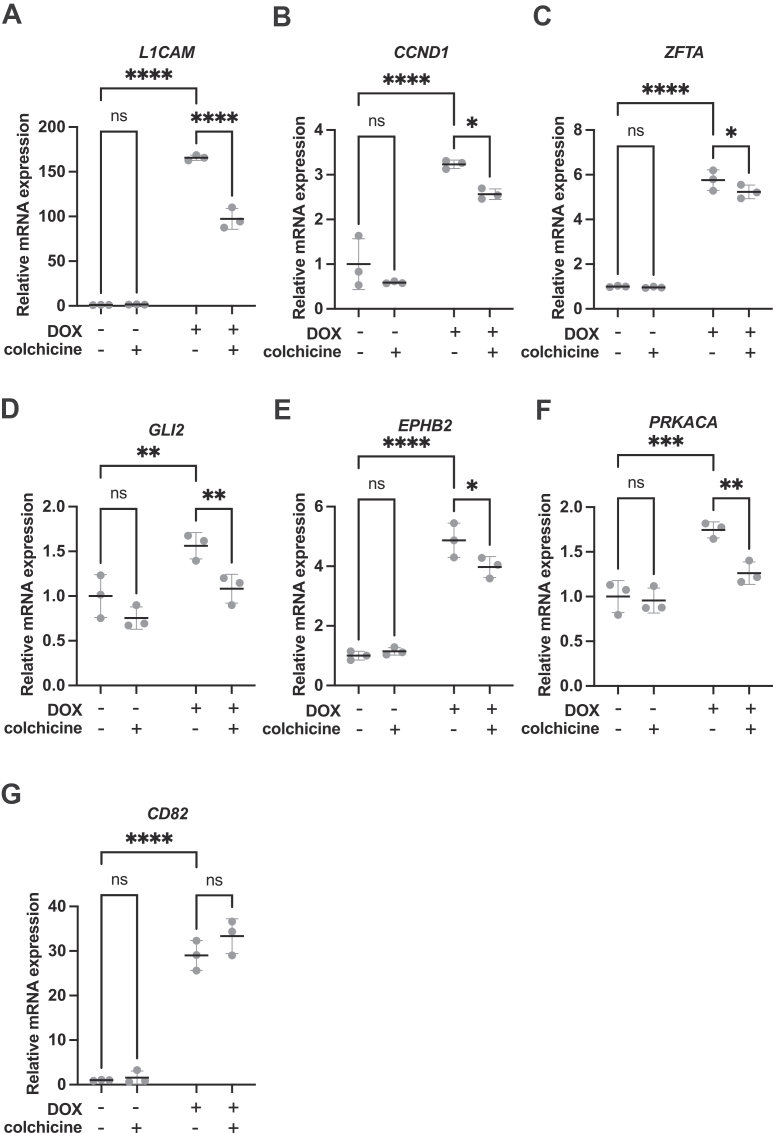
**Effect of colchicine on the expression of endogenous NF-κB responsive genes and *ZFTA* (*A–G*).** Relative expression of *L1CAM* (*A*), *CCND1* (*B*), *ZFTA* (*C*), *GLI2* (*D*), *EPHB2* (*E*), *PRKACA* (*F*), and *CD82* (*G*) mRNAs in 6E8 cells cultured overnight in the absence (−) or presence (+) of 300 ng/ml doxycycline (DOX) and 0.10 μM colchicine. The mRNA levels of the doxycycline control were set to 1. The data are presented as the mean ± SD. Each point on the graph represents the relative mRNA expression level of an individual sample. n = 3 biological replicates each. Statistical analysis was done using a one-way ANOVA followed by an uncorrected Fisher's LSD test. ns, not significant; ∗*p* < 0.05; ∗∗*p* < 0.01; ∗∗∗*p* < 0.001; ∗∗∗∗*p* < 0.0001.

To validate the screening results, we examined the effect of colchicine on the upregulation of endogenous gene expression induced by ZFTA-RELA using 6E8 cells. As previously reported (9, 14, 15, 23), the expression of ZFTA-RELA induced by addition of doxycycline to the 6E8 cells resulted in increased expression of *CCND1* and *L1CAM* (Fig. 2, *A* and *B*). Colchicine attenuated the upregulated expression of these genes (Fig. 2, *A* and *B*); for example, colchicine reduced ZFTA-RELA-induced *L1CAM* expression by approximately 41% and *CCND1* expression by approximately 21%. Overexpression of ZFTA-RELA, but not RELA, results in the upregulation of *ZFTA* expression (23, 24). In addition, ZFTA-fusion proteins, specifically ZFTA-RELA, ZFTA-MAML2, and ZFTA-NCOA2, promote the expression of common target genes, such as *GLI2*, *EPHB2*, *PRKACA*, and *CD82* (9). Colchicine partially inhibited ZFTA-RELA-induced upregulation of most of these target genes, with reductions of approximately 9% for *ZFTA*, 31% for *GLI2*, 18% for *EPHB2*, and 28% for *PRKACA* (Fig. 2, *C*–*F*). In contrast, *CD82*, although robustly induced by ZFTA-RELA, was not reduced by colchicine (Fig. 2*G*). These results suggest that colchicine inhibits the processes that result in transcriptional activation by ZFTA-RELA, including its nuclear translocation, although this inhibition is not uniform across all responsive genes.

### Reduction of ZFTA-RELA nuclear localization by targeting microtubule–dynein machinery

**Figure 3:**
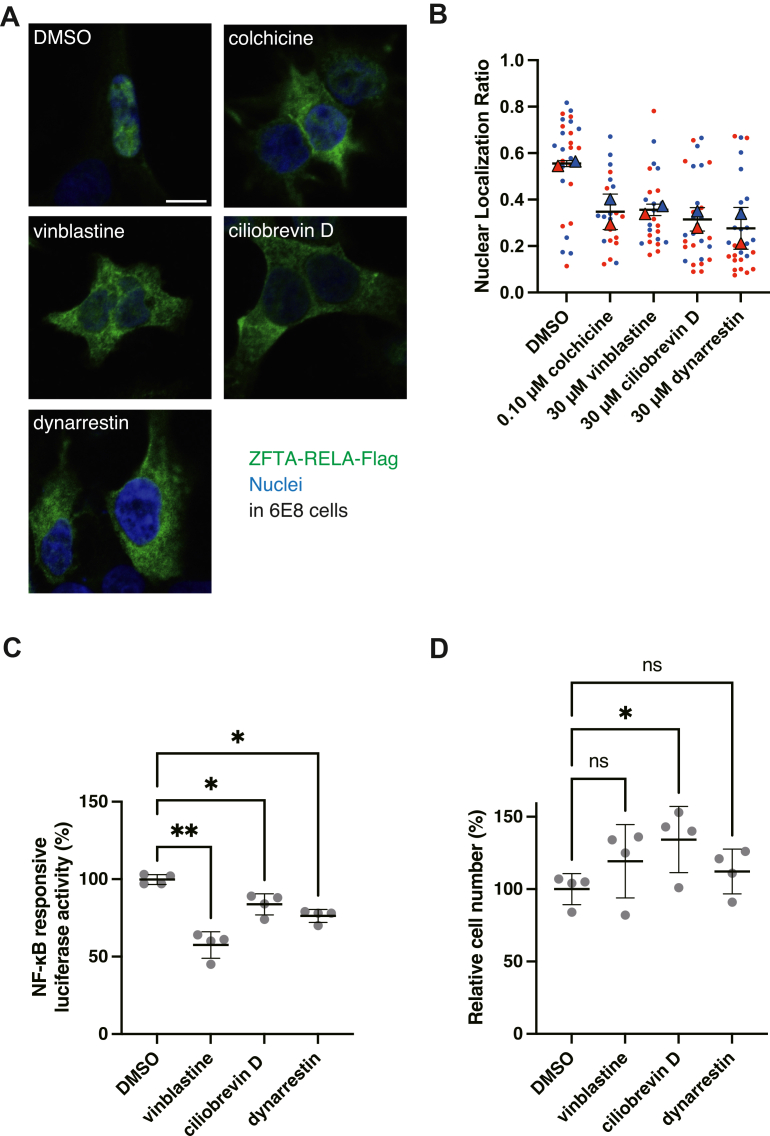
**Vinblastine, ciliobrevin D, and dynarrestin treatment results in a reduction in NF-κB-responsive luciferase activity and the nuclear localization ratio of ZFTA-RELA in 6E8 cells.** (*A* and *B*) Representative confocal images (*A*) and quantification of the nuclear localization of ZFTA-RELA-Flag (*B*) in 6E8 cells. ZFTA-RELA-Flag (*green*), nuclei (*blue*). DMSO served as the solvent control. The scale bar represents a length of 10 μm. Data are presented as a SuperPlot of two independent experiments: each small symbol represents the nuclear localization ratio of ZFTA-RELA-Flag in an individual cell, color-coded by experiment, and each large symbol represents the per-experiment mean (biological replicate). The total numbers of cells analyzed were 27 (DMSO), 23 (colchicine), 24 (vinblastine), 28 (ciliobrevin D), and 28 (dynarrestin). (*C* and *D*) NF-κB-responsive luciferase activity (*C*) and the relative viable cell number (*D*) in 6E8 cells. DMSO served as the solvent control. The mean reporter activity (*C*) and cell number (*D*) in DMSO control cells were set to 100%. The data presented are the mean ± SD. Each data point on the graph represents the relative reporter activity (*C*) and cell number (*D*) of an individual sample. The number of samples (n) was four for each data point (biological replicates). Statistical analysis was done using a one-way ANOVA with Tukey’s *post hoc* test. ns, not significant; ∗*p* < 0.05; ∗∗*p* < 0.01.

Microtubule destabilization inhibits the activation of the NF-κB pathway and the nuclear translocation of RELA in neuroblastoma cells (27). Therefore, we determined whether ZFTA-RELA uses a similar microtubule-dependent transport mechanism for its nuclear localization. In 6E8 cells treated with DMSO and doxycycline (DMSO control), ZFTA-RELA-Flag was localized to the nucleus (Figs. 3*A*, S1). In contrast, the nuclear localization of ZFTA-RELA-Flag was reduced in 6E8 cells treated with 0.10 μM colchicine, as shown in the immunostaining images (Fig. 3*A*, S1) and confirmed by quantification of the nuclear localization ratio (Fig. 3*B*). The results suggest that an intact microtubule network contributes to the nuclear localization of ZFTA-RELA.

Dynein is a motor protein that moves along microtubules, facilitating the nuclear transport of RELA (28, 29). To determine whether microtubule polymerization and dynein are also involved in the nuclear localization of ZFTA-RELA and the upregulation of ZFTA-RELA-mediated gene expression, we treated 6E8 cells with another microtubule polymerization inhibitor, vinblastine, and two inhibitors of dynein, ciliobrevin D and dynarrestin (32, 33, 34). We observed a partial reduction in the nuclear localization of ZFTA-RELA-Flag in 6E8 cells, with reductions of approximately 38%, 36%, 44%, and 52% upon treatment with 0.10 μM colchicine, 30 μM vinblastine, 30 μM ciliobrevin D, and 30 μM dynarrestin, respectively (Fig. 3, *A* and *B*). Moreover, treatment with vinblastine, ciliobrevin D, and dynarrestin resulted in a partial inhibition of the doxycycline-induced increase in NF-κB-responsive luciferase activity in 6E8 cells, showing reductions of 42%, 16%, and 24%, respectively (Fig. 3*C*). Importantly, these inhibitors did not reduce the number of viable cells in 6E8 cells at the concentration used (Fig. 3*D*).

Cytoplasmic dynein contributes not only to cargo transport but also to the maintenance of radial microtubule organization in interphase cells, and prolonged dynein inhibition has been reported to disrupt the microtubule network (35). Therefore, the reduction in ZFTA-RELA nuclear localization observed under ciliobrevin D and dynarrestin treatment could be attributable to disruption of the microtubule network rather than to direct inhibition of dynein-mediated transport. To address this possibility, we examined microtubule network integrity by α-tubulin staining in 6E8 cells treated with these dynein inhibitors overnight (Fig. S2). In cells treated with 0.10 μM colchicine, the microtubule network was markedly disorganized compared with the DMSO-treated control cells. In contrast, in cells treated with 30 μM ciliobrevin D or 30 μM dynarrestin, the α-tubulin staining patterns were comparable to those of the DMSO control, with filamentous microtubule networks preserved throughout the cytoplasm. These results suggest that the reduction in ZFTA-RELA nuclear localization caused by ciliobrevin D and dynarrestin (Fig. 3, *A* and *B*) is not likely due to microtubule network disruption. Altogether, these results suggest that microtubule polymerization and dynein contribute to the nuclear localization of ZFTA-RELA, which in turn, upregulates ZFTA-RELA-responsive gene expression; however, the incomplete reduction observed upon pharmacological inhibition suggests that microtubule-independent mechanisms also participate in its nuclear localization.

### Reduction in the nuclear localization of ZFTA-RELA by colchicine is dependent on the RELA region of ZFTA-RELA

**Figure 4:**
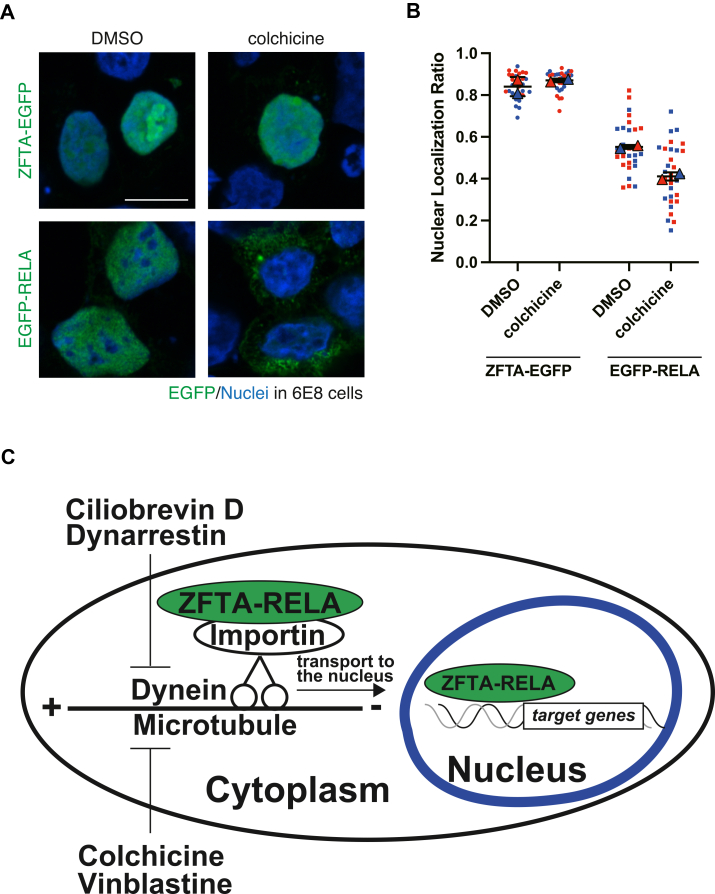
**Reduction of the nuclear localization of ZFTA-RELA is mediated through the RELA, but not ZFTA region.** (*A* and *B*) Representative confocal images (*A*) and quantification of the nuclear localization (*B*) of ZFTA-EGFP and EGFP-RELA in 6E8 cells. (*A*) ZFTA-EGFP and EGFP-RELA (*green*), nuclei (*blue*). DMSO served as the solvent control. The scale bar represents a length of 10 μm. (*B*) Data are presented as a SuperPlot of two independent experiments: each small symbol represents the nuclear localization ratio of an individual cell, color-coded by experiment, and each large symbol represents the per-experiment mean (biological replicate). A total of 30 cells were analyzed per group. (*C*) Proposed model for colchicine-sensitive nuclear transport of ZFTA-RELA.

Although RELA is transported into the nucleus along microtubules *via* importins (27, 36), the ZFTA region of ZFTA-RELA also contains its own nuclear localization mechanism (7, 9, 21, 23). We determined whether the inhibitory effects of microtubule destabilization on the nuclear localization of ZFTA-RELA are mediated by the ZFTA or RELA regions. By replacing the ZFTA and RELA regions of ZFTA-RELA with enhanced green fluorescent protein (EGFP), we prepared plasmids encoding ZFTA-EGFP (exons one and two of *ZFTA* were fused with *egfp*) and EGFP-RELA (exons from 2 to 11 of *RELA* were fused with *egfp*). These were expressed in 6E8 cells, and their subcellular distribution was analyzed. Consistent with previous reports (21, 23), ZFTA-EGFP predominantly accumulated in the nucleus, and the nuclear accumulation pattern remained unchanged following treatment with colchicine (Figs. 4*A*, S3). In contrast, EGFP-RELA localized to the nucleus in TNF-α-stimulated 6E8 cells as demonstrated for full-length RELA (27); however, this nuclear localization was reduced by colchicine treatment by approximately 24% (Figs. 4*B*, S3). These results suggest that the reduction in ZFTA-RELA nuclear localization by microtubule destabilization occurs through impairment of the nuclear transport mechanism mediated by the RELA region of the fusion protein (Fig. 4*C*).

### Colchicine-mediated attenuation of nuclear localization of ZFTA-NCOA2 and ZFTA-MKL2

**Figure 5:**
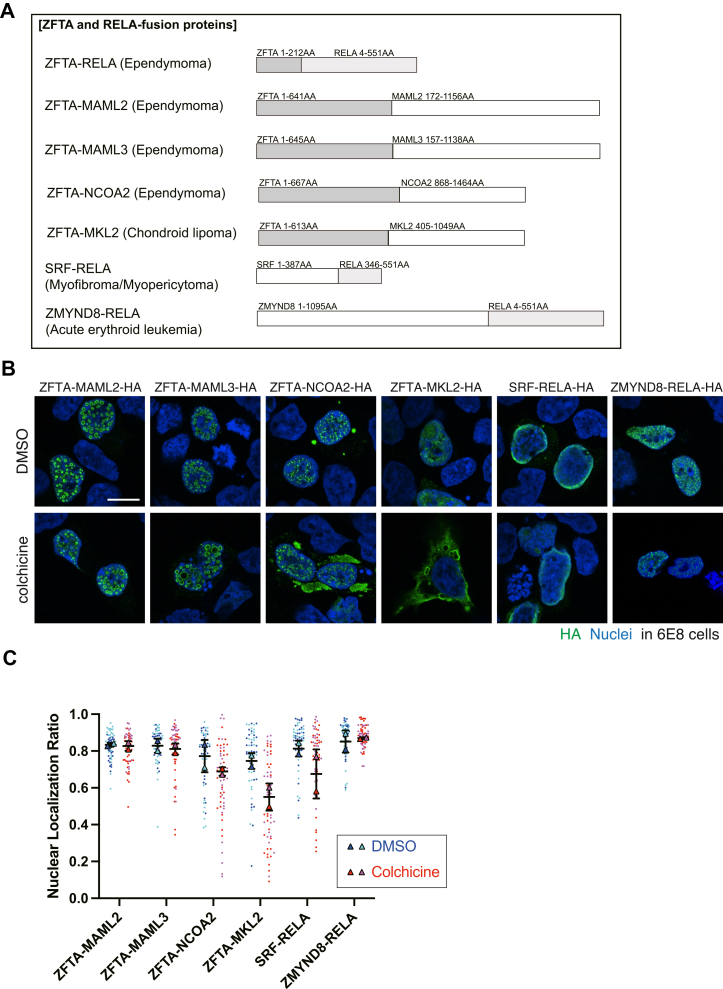
**Differential sensitivity of ZFTA and RELA-fusion proteins to colchicine-mediated inhibition of nuclear localization.** (*A*) Schematic representation of the ZFTA and RELA-fusion proteins examined in this study, showing the retained regions and fusion boundaries (amino acid numbers) of ZFTA and of each fusion partner. (*B*) Representative confocal images of 6E8 cells transiently expressing HA-tagged ZFTA or RELA-fusion proteins (ZFTA-MAML2-HA, ZFTA-MAML3-HA, ZFTA-NCOA2-HA, ZFTA-MKL2-HA, SRF-RELA-HA, and ZMYND8-RELA-HA) cultured overnight in the absence (DMSO) or presence of 0.10 μM colchicine. HA-tagged fusion proteins (*green*), nuclei (*blue*). Scale bar, 10 μm. (*C*) Quantification of nuclear localization ratio for each fusion protein in the absence (DMSO) or presence of 0.10 μM colchicine. Data are presented as a SuperPlot of two independent experiments: each small symbol represents the nuclear localization ratio of an individual cell, and each large symbol represents the per-experiment mean (biological replicate). Colors denote the treatment (DMSO, *blue*; *colchicine*, *red*), and the two color shades and the two large symbols within each treatment correspond to the two independent experiments. A total of 60 cells were analyzed per group, except for ZMYND8-RELA with colchicine treatment (61 cells).

Besides the major ZFTA-RELA fusion, ZFTA forms oncogenic fusion proteins with various transcriptional regulators, including MAML2/3 and NCOA2 in ST-EPN-ZFTA, all of which constitutively localize to the nucleus to promote aberrant gene expression (9). Similarly, ZFTA participates in fusion events with MKL2 in chondroid lipomas, whereas RELA fuses with SRF in myofibroma/myopericytoma and with ZMYND8 in acute erythroid leukemia (12, 13, 37, 38); however, the subcellular localization of these fusion proteins remains unknown. Because of our finding that the microtubule–dynein machinery mediates ZFTA-RELA nuclear localization, we determined whether this transport mechanism is shared by various ZFTA- and RELA-fusion proteins by examining their subcellular distribution patterns and responsiveness to colchicine treatment (Fig. 5*A*). In 6E8 cells, immunocytochemical analysis revealed the predominant nuclear localization of the HA-tagged ZFTA and RELA-fusion proteins examined: ZFTA-NCOA2-HA, ZFTA-MAML2-HA, ZFTA-MAML3-HA, ZFTA-MKL2-HA, SRF-RELA-HA, and ZMYND8-RELA-HA (Figs. 5, *A*–*C* and S4). Interestingly, ZFTA-MAML2-HA and ZFTA-MAML3-HA displayed a punctate nuclear pattern similar to that reported for full-length MAML3 in neuroendocrine tumor cells (39). This suggests that the MAML region may contribute to this distinctive subnuclear distribution pattern. Notably, colchicine treatment reduced the nuclear localization of ZFTA-NCOA2-HA, ZFTA-MKL2-HA, and SRF-RELA-HA (by approximately 11%, 26%, and 17%, respectively), with little to no change for ZFTA-MAML2-HA, ZFTA-MAML3-HA, and ZMYND8-RELA-HA (Figs. 5, *B* and *C*, S4). The results indicate that the microtubule plays an important role in the nuclear localization of specific ZFTA-fusion proteins, with a requirement for this transport mechanism varying depending on the fusion partner and length of the ZFTA region.

### Colchicine reduces gene expression induced by ZFTA-NCOA2 and ZFTA-MKL2

**Figure 6:**
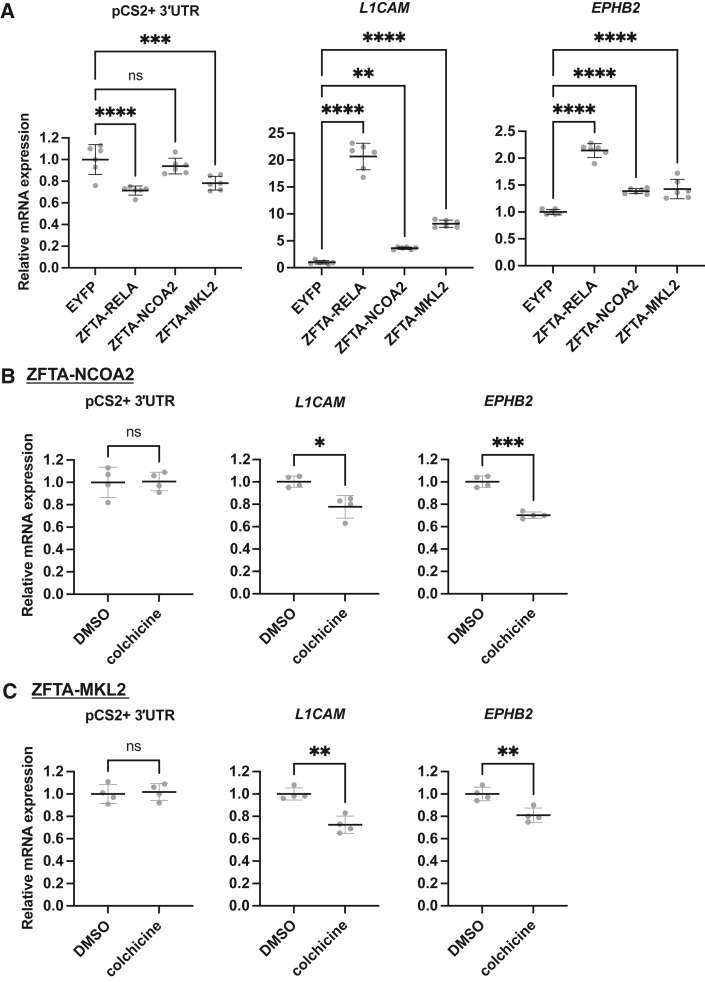
**Colchicine attenuates gene expression induced by ZFTA-NCOA2 and ZFTA-MKL2.** (*A*) Relative mRNA expression levels of transfected plasmids (pCS2+ 3′UTR), *L1CAM*, and *EPHB2* in 6E8 cells transiently transfected with pCS2+ plasmids encoding EYFP, ZFTA-RELA-HA, ZFTA-NCOA2-HA, or ZFTA-MKL2-HA. The average mRNA levels in *eyfp*-transfected cells were set to 1. Statistical analysis was performed using a one-way ANOVA with Dunnett's multiple comparisons test. n = 6 biological replicates each. (*B* and *C*) The effect of 0.10 μM colchicine on gene expression in 6E8 cells transfected with ZFTA-NCOA2-HA (*B*) or ZFTA-MKL2-HA (*C*). Relative mRNA expression levels of the transfected plasmids (pCS2+ 3′UTR), *L1CAM*, and *EPHB2*. Data in all panels are presented as the mean ± SD. Each data point represents the relative mRNA expression level of an individual sample. n = 4 biological replicates each. Statistical analysis was performed using an unpaired two-tailed Student’s *t* test. ns, not significant; ∗*p* < 0.05; ∗∗*p* < 0.01; ∗∗∗*p* < 0.001; ∗∗∗∗*p* < 0.0001.

After establishing that colchicine attenuates the nuclear localization of ZFTA-NCOA2 and ZFTA-MKL2, we determined whether colchicine also suppresses gene expression induced by these fusion proteins. First, we identified genes whose expression is upregulated by ZFTA-NCOA2 or ZFTA-MKL2. We transiently transfected 6E8 cells with pCS2+ plasmids encoding enhanced yellow fluorescent protein (EYFP), ZFTA-RELA-HA, ZFTA-NCOA2-HA, or ZFTA-MKL2-HA (Fig. 5*A*) and measured their expression by qPCR using the primers targeting the common 3′UTR sequence shared by all plasmids. The relative mRNA levels (mean ± SD) of *eyfp*, *ZFTA-RELA*, *ZFTA-NCOA2*, and *ZFTA-MKL2* were 1.0 ± 0.14, 0.71 ± 0.04, 0.94 ± 0.07, and 0.78 ± 0.07, respectively (Fig. 6*A*), indicating that the fusion constructs were expressed at levels comparable to or lower than that of *eyfp*. Consistent with previous findings in human tumors and cultured cells (9), ZFTA-RELA and ZFTA-NCOA2 increased *L1CAM* and *EPHB2* expression in 6E8 cells. We also found that ZFTA-MKL2 upregulates the expression of these genes (Fig. 6*A*).

Next, we determined the effect of colchicine treatment on the expression of these genes. Colchicine treatment did not alter the expression of transfected *ZFTA-NCOA2-HA* or *ZFTA-MKL2-HA*; however, it significantly attenuated the ZFTA-NCOA2-induced upregulation of *L1CAM* and *EPHB2* by approximately 22% and 30%, respectively (Fig. 6*B*), and the ZFTA-MKL2-induced upregulation of *L1CAM* and *EPHB2* by approximately 28% and 19%, respectively (Fig. 6*C*). These results indicate that colchicine treatment partially attenuates the transcriptional activation of target genes induced by ZFTA-NCOA2 and ZFTA-MKL2 in cultured cells, although the magnitude of suppression was modest.

### Identification of sequences with microtubule-dependent transport activity in ZFTA-fusion proteins

**Figure 7:**
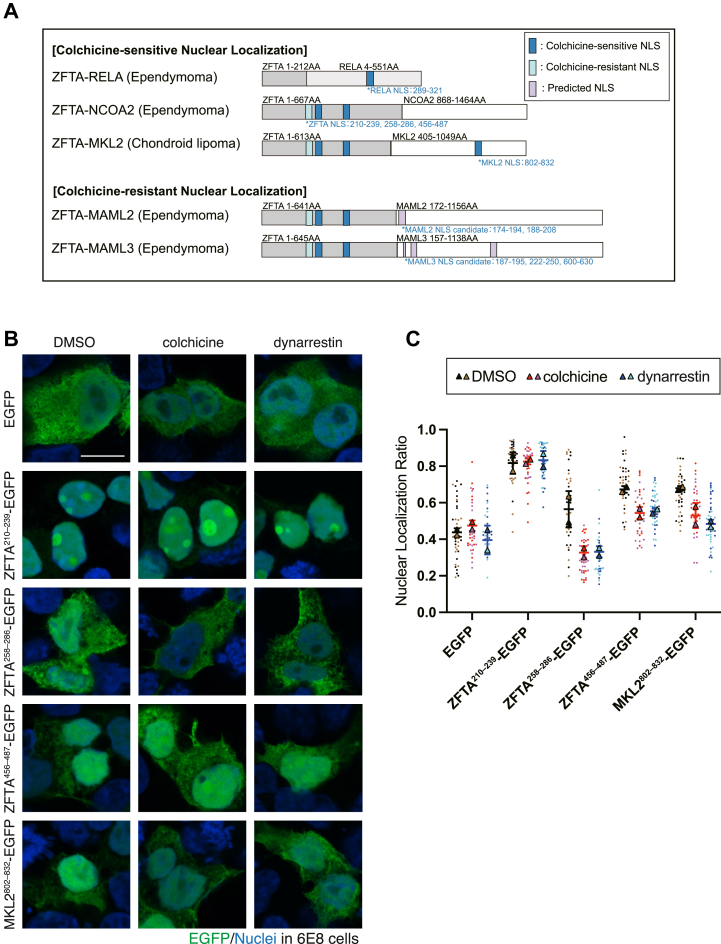
**Identification of microtubule-dependent NLSs in ZFTA-fusion proteins.** (*A*) Schematic representation of the ZFTA-fusion proteins, grouped according to the colchicine sensitivity of their nuclear localization (*top*, colchicine-sensitive: ZFTA-RELA, ZFTA-NCOA2, and ZFTA-MKL2; *bottom*, colchicine-resistant: ZFTA-MAML2 and ZFTA-MAML3). The amino acid boundaries of each fusion partner are indicated. NLS sequences are color-coded as follows: *dark blue*, colchicine-sensitive NLSs; *light teal*, the colchicine-resistant NLS; *purple*, putative NLSs predicted by cNLS Mapper within the MAML2 and MAML3 regions that were not individually tested. (*B*) Representative confocal images of 6E8 cells transiently expressing EGFP alone or EGFP fusion proteins containing individual cNLS sequences (ZFTA^210–239^-EGFP, ZFTA^258–286^-EGFP, ZFTA^456–487^-EGFP, and MKL2^802–832^-EGFP), cultured overnight in the presence of 0.3% DMSO (control), 0.10 μM colchicine, or 30 μM dynarrestin. EGFP signal (*green*), nuclei (*blue*). Scale bar, 10 μm. (*C*) Quantification of the nuclear localization ratio for each EGFP fusion protein under the indicated treatments. Data are presented as a SuperPlot of two independent experiments: each small symbol represents the nuclear localization ratio of an individual cell, and each large symbol represents the per-experiment mean (biological replicate). Colors denote the treatment (DMSO, *black*; *colchicine*, *red*; dynarrestin, *blue*), and the two color shades and the two large symbols within each treatment correspond to the two independent experiments. The total numbers of cells analyzed were 36 to 43 (DMSO), 35 to 39 (colchicine), and 36 to 41 (dynarrestin).

We considered how other colchicine-sensitive ZFTA-fusion proteins, namely ZFTA-NCOA2 and ZFTA-MKL2, which lack the RELA component, achieve microtubule-dependent nuclear localization. NCOA2 and MKL2 have known NLS-containing domains in their full-length forms. NCOA2 contains a basic helix-loop-helix/Per-ARNT-SIM (bHLH-PAS) domain, whose NLS activity was reported in the related family member NCOA3/SRC-3 (40, 41), and MKL2 contains N-terminal RPEL motifs that mediate the importin α/β interaction in the related MKL1 (42, 43, 44); however, these NLS regions are not involved in ZFTA-NCOA2 or ZFTA-MKL2 (9, 12, 13). Notably, ZFTA-NCOA2 and ZFTA-MKL2 contain longer ZFTA regions compared with ZFTA-RELA (6, 7, 8, 9, 10, 12, 13), suggesting that these extended ZFTA regions may harbor alternative NLS sequences that are responsible for their nuclear localization. To test this hypothesis, we performed cNLS mapper analysis on the complete sequences of the ZFTA- and RELA-fusion proteins, including ZFTA-NCOA2 and ZFTA-MKL2 (Fig. 7*A*). We identified four putative cNLS sequences: three within the extended ZFTA region (residues 210–239, 258–286, and 456–487) and one within the MKL2 region (residues 802–832, corresponding to residues 1011–1041 of ZFTA-MKL2) (Fig. 7*A*). cNLS Mapper also predicted candidate NLS sequences within the MAML2 and MAML3 regions of the colchicine-resistant fusion proteins ZFTA-MAML2 and ZFTA-MAML3 (Fig. 7*A*). Because these fusions retained nuclear localization upon colchicine treatment (Fig. 5), we focused our functional validation on the four candidate sequences above and considered the MAML2/3 predictions in the Discussion. To determine whether these predicted cNLS sequences are sufficient to confer microtubule-dependent nuclear localization, we generated EGFP fusion constructs containing each of these sequences individually (ZFTA^210–239^-EGFP, ZFTA^258–286^-EGFP, ZFTA^456–487^-EGFP, and MKL2^802–832^-EGFP) and determined their subcellular localization in 6E8 cells. Under control conditions (0.3% DMSO treatment), all four cNLS-EGFP fusion proteins exhibited approximately 1.3- to 1.9-fold higher nuclear localization ratios compared with EGFP alone (Figs. 7, *B* and *C*, S5). This confirms that the identified sequences are sufficient to confer nuclear localization. To determine whether these sequences are transported through the microtubule–dynein machinery, we treated the transfected 6E8 cells with 0.10 μM colchicine or 30 μM dynarrestin. These agents reduced the nuclear localization of ZFTA^258–286^-EGFP (by approximately 48% and 47% with colchicine and dynarrestin, respectively), ZFTA^456–487^-EGFP (by approximately 23% and 19%, respectively), and MKL2^802–832^-EGFP (by approximately 13% and 25%, respectively) (Fig. 7, *B* and *C*, S5). In contrast, ZFTA^210–239^-EGFP showed only minimal changes upon either treatment (approximately 3% and 0% reduction, respectively). The concordant reduction by both colchicine and dynarrestin suggests that these three sequences (ZFTA^258–286^, ZFTA^456–487^, and MKL2^802–832^) can mediate nuclear localization through a microtubule–dynein-dependent pathway.

## Discussion

Because targeting the ZFTA-fusion proteins represents a promising therapeutic strategy for ST-EPN-ZFTA (24), we conducted a chemical screen to identify inhibitors of aberrant gene expression induced by ZFTA-RELA. The analysis revealed that colchicine, a microtubule polymerization inhibitor, attenuated the aberrant gene expression induced by ZFTA-RELA. We found that ZFTA-RELA uses the microtubule–dynein machinery for nuclear transport, similar to wild-type RELA (26, 27, 28), and this transport is mediated through the RELA region of the fusion protein. However, pharmacological inhibition of microtubule polymerization or dynein did not completely abolish the nuclear translocation of ZFTA-RELA or NF-κB-responsive luciferase activity, suggesting the existence of additional microtubule-independent transport mechanisms. This alternative pathway may involve the ZFTA region, as previously reported for ZFTA region-dependent nuclear localization (7, 9, 21, 23). Importantly, we also discovered that this microtubule-dependent nuclear transport mechanism is conserved across other ZFTA-fusion proteins, including ZFTA-NCOA2 (ST-EPN-ZFTA) and ZFTA-MKL2 (chondroid lipomas). Therefore, we not only elucidated a part of the molecular mechanisms underlying the pathogenesis of ZFTA-fusion-driven tumors but also propose microtubule–dynein machinery as a potential treatment target for these malignancies.

In the present study, we assessed the intracellular localization of various ZFTA- and RELA-fusion proteins and determined the effects of microtubule polymerization and dynein inhibition on their localization. Although ZFTA-RELA, ZFTA-MAML2/3, and ZFTA-NCOA2 were previously shown to be nuclear-localized (9), the localization of ZFTA-MKL2, SRF-RELA, and ZMYND8-RELA remains unknown. Our results indicate that these fusion proteins localize to the nucleus, suggesting their potential involvement in transcriptional regulation. Notably, we observed distinct subnuclear distribution patterns among these proteins. ZFTA-MAML2 and ZFTA-MAML3 exhibited a punctate nuclear pattern, reminiscent of MAML3 in neuroendocrine tumors (39). Similarly, ZFTA-NCOA2 showed a punctate nuclear distribution, consistent with the focal pattern observed for NCOA2 in association with promyelocytic leukemia (PML) bodies (45). These results are consistent with a recent study showing that ZFTA-RELA forms nuclear condensates that are essential for oncogenic transcription (46). These distinct subnuclear patterns among ZFTA-fusion proteins suggest variant-specific mechanisms of localization and transcriptional regulation that warrant further studies to elucidate ZFTA-fusion-driven tumorigenesis.

Our study revealed that the microtubule–dynein machinery mediates the nuclear transport of specific ZFTA-fusion proteins. Although ZFTA-RELA uses its RELA region containing an established cNLS (26, 27, 28), ZFTA-NCOA2 and ZFTA-MKL2 likely utilize alternative sequences within the extended ZFTA or partner regions. Notably, we identified a candidate cNLS sequence within the MKL2 region (MKL2^802–832^) distinct from the RPEL motifs that mediate nuclear import in related MKL family members (42, 43, 44). When fused to EGFP, three sequences (ZFTA^258–286^, ZFTA^456–487^, and MKL2^802–832^) displayed microtubule-dependent nuclear localization, whereas ZFTA^210–239^ showed microtubule-independent activity. Interestingly, ZFTA-MAML2/3 fusion proteins maintained nuclear localization upon colchicine treatment, despite containing colchicine-sensitive putative cNLSs (ZFTA^258–286^ and ZFTA^456–487^). This suggests that the predicted NLS in the MAML2/3 regions (Fig. 7*A*) may override the colchicine-sensitive transport pathway. The redundancy in nuclear transport pathways, which use both microtubule-dependent and -independent mechanisms, highlights the complexity of fusion protein subcellular localization and the importance of mechanistic studies to further understand their nuclear import.

How nuclear import is achieved through colchicine-insensitive NLS sequences, such as ZFTA^210–239^ and the predicted NLS candidates in the MAML2/3 regions (Fig. 7), remains an open question. Several mechanisms may account for microtubule-independent nuclear import mediated by these sequences. First, classical importin α/β-mediated transport does not inherently require microtubule-based cytoplasmic delivery; NLS sequences with sufficiently high importin affinity may reach the nuclear pore complex through diffusion alone (47). Second, these sequences may harbor non-classical signals recognized directly by alternative members of the karyopherin β superfamily (48). Third, piggybacking through interaction with endogenous nuclear-targeted proteins may contribute (48). Identification of the specific import receptors and signal sequences responsible for these colchicine-insensitive NLS-mediated transport pathways will be an important direction for future studies.

Although cytoplasmic dynein contributes to microtubule organization in interphase cells (35), the microtubule network remained largely intact in 6E8 cells treated overnight with ciliobrevin D or dynarrestin (Fig. S2). Our results suggest that disruption of microtubule architecture is unlikely to be the principal cause of the reduced ZFTA-RELA nuclear localization observed under these conditions. We cannot, however, fully exclude a partial contribution from subtle perturbations of microtubule organization that are not detectable by immunofluorescence staining. Nevertheless, the magnitude of inhibition differed between microtubule polymerization inhibitors and dynein inhibitors for reporter activity (Fig. 3*C*), whereas the effects on nuclear localization ratio were comparable across all inhibitors tested (Fig. 3*B*). This dissociation suggests that the difference in transcriptional inhibition is not fully accounted for by differences in ZFTA-RELA nuclear accumulation alone. One possible interpretation is that colchicine and vinblastine exert additional effects on transcriptional activity beyond reducing nuclear import—for example, by disrupting microtubule-dependent processes involved in transcriptional regulation, chromatin organization, or NF-κB pathway activation that are independent of cargo transport. Alternatively, the currently available dynein inhibitors, ciliobrevin D and dynarrestin, have recognized limitations in target specificity and cellular potency (33, 34), and off-target effects of these agents—independent of dynein inhibition itself—may influence transcriptional readouts. At present, we cannot fully distinguish between these possibilities, and the relationship between ZFTA-RELA nuclear localization and its transcriptional activity warrants further mechanistic investigation.

Among the RELA-containing fusions from other tumor types (37, 38), SRF-RELA exhibited a reduction in nuclear localization upon microtubule polymerization inhibition, whereas ZMYND8-RELA showed little to no reduction. This differential sensitivity may reflect their distinct structural features. SRF-RELA lacks the colchicine-sensitive NLS within the RELA region (residues 289–321) (36, 37), but includes established and predicted NLS (residues 95–100 and 141–168 of SRF, respectively) (49); its nuclear localization may therefore be governed by these SRF-derived signals rather than the RELA-derived NLS. In contrast, ZMYND8-RELA contains both the RELA-derived colchicine-sensitive NLS and a predicted NLS (residues 34–70), yet remained colchicine-resistant, suggesting that this predicted NLS provides microtubule-independent nuclear transport. Our results highlight the complex interplay between fusion protein structure and nuclear transport mechanisms, with significant implications for understanding ST-EPN pathogenesis. Further structural and biochemical analyses are warranted to fully characterize these distinct nuclear transport mechanisms and their contribution to oncogenic transcription.

The identification of microtubule–dynein machinery as an important mediator of ZFTA-fusion protein nuclear transport in the present study provides a rational basis for therapeutic intervention. Notably, the nuclear level of ZFTA-RELA appears to require tight regulation: a recent study showed that excessive nuclear accumulation caused by Exportin-1 inhibition suppresses ST-EPN-ZFTA in patient-derived models (25). Together with our finding that lowering nuclear ZFTA-RELA *via* import inhibition attenuates its transcription, these observations indicate that the tumor is vulnerable to disruption of ZFTA-RELA nucleocytoplasmic homeostasis, positioning this trafficking axis as a therapeutic target. Importantly, although colchicine did not attenuate all ZFTA-RELA-responsive genes to the same extent (Fig. 2), *GLI2* and *EPHB2*—key oncogenic downstream genes of ZFTA-fusion proteins (9)—were among those attenuated, indicating that microtubule inhibition can lower the expression of tumorigenically relevant genes. However, not all microtubule-targeting agents are likely to be suitable for this tumor type: paclitaxel, a widely used microtubule-stabilizing chemotherapeutic agent, has been reported to activate the NF-κB pathway (26), which would counteract the therapeutic goal of suppressing oncogenic transcription driven by ZFTA-RELA.

Colchicine is primarily used for the management of gout and familial Mediterranean fever because of its anti-inflammatory properties (31). Nevertheless, it is not commonly used for cancer treatment because of its toxicity (50, 51, 52). Although our results indicate that colchicine inhibits the nuclear transport of ZFTA-RELA, the development of effective and less toxic colchicine derivatives is an important step. Consistent with previous reports (50, 52, 53), the C7-derivatives showed a minimal reduction in NF-κB inhibitory activity, whereas the C10-derivatives demonstrated a marked decrease in inhibitory activity. Considering that the C10-methoxy group of colchicine is located on the binding side with tubulin (54, 55), C10-derivatives may exhibit reduced efficiency in binding to tubulin because of steric hindrance. A more promising route for the synthesis of colchicine derivatives would be the modification of C7, because it is on the opposite side of the tubulin binding side (54, 55), and the NF-κB inhibitory activity was not significantly reduced for the C7-derivatives. Moreover, the nitrogen at C7 is indispensable for the recognition of colchicine derivatives by P-glycoprotein, which extrudes drugs from the cell (56). These structure-activity relationships provide a rational framework for developing safer colchicine derivatives that retain potent inhibitory effects on ZFTA-fusion protein nuclear transport, while minimizing systemic toxicity.

A limitation of the present study is that all experiments were performed in the HEK293-derived 6E8 cell line. We attempted to evaluate the effect of colchicine in EP1NS cells, a human ependymoma-derived cell line (57); however, these cells exhibited high sensitivity to colchicine, resulting in cell death that precluded analysis of nuclear localization and gene expression (data not shown). Cell-type-specific differences in microtubule dynamics and the expression profiles of import receptors, including importins and karyopherins, may influence the efficiency and pathway utilization of fusion protein nuclear transport. Validation of these findings in ependymoma-derived models with differing colchicine sensitivity remains an important future direction.

In conclusion, we identified the microtubule–dynein machinery as an important mediator of ZFTA-RELA nuclear transport and showed that disrupting this pathway attenuates oncogenic transcription. The differential sensitivity of fusion proteins to microtubule inhibition reflects their distinct nuclear transport mechanisms. Although ZFTA-RELA, ZFTA-NCOA2, and ZFTA-MKL2 depend on microtubule-mediated transport, at least in part, the other fusion proteins examined use alternative pathways that remain unclear. This mechanistic insight provides a molecular basis for targeting ST-EPN-ZFTA through the inhibition of microtubule-dependent nuclear transport.

## Experimental procedures

### Cell culture

The 6E8 cell line was established from a commercially available NF-κB Reporter (Luc) HEK293 Recombinant Cell Line (BPS Bioscience, Cat. #60650) (14). The parental cell line was obtained directly from the supplier and has undergone quality control, including *mycoplasma* testing by them. Short tandem repeat profiling of the 6E8 cell line has not been independently performed. The 6E8 cells express the most prevalent ZFTA-RELA variant (ZFTA-RELA^FUS1^) in a doxycycline concentration-dependent manner (7). The cells were cultured in Dulbecco’s modified Eagle medium (Nacalai Tesque) supplemented with 4.5 g/L glucose, 110 mg/L sodium pyruvate, 10% (v/v) fetal bovine serum (Biowest), and antibiotic-antimycotic solution (Nacalai Tesque), hereafter referred to as the culture media, at 37 °C in a 5% CO_2_ atmosphere. The expression of ZFTA-RELA containing a Flag tag at its C-terminus (ZFTA-RELA-Flag) in 6E8 cells was induced by the addition of 300 ng/ml doxycycline (Clontech) to the culture medium.

### Compounds

The DDI core library was provided by the Drug Discovery Initiative (DDI) of the University of Tokyo (https://www.ddi.f.u-tokyo.ac.jp/en/). The chemical compounds used in this study are listed in Table S2.

### Relative viable cell counts and luciferase assays

Relative viable cell numbers were quantified using Cell Count Reagent SF (Nacalai Tesque) based on the manufacturer’s instructions, using a TriStar LB 941 instrument (Berthold). The luciferase assay was performed as described previously (14, 15) using TriStar LB 941 (Berthold) in 100 mM Tris-HCl (pH 7.0, Nacalai Tesque), 5 mM MgCl_2_ (FUJIFILM), 250 μM coenzyme A trilithium salt (Oriental Yeast), 150 μM adenosine triphosphate (Nacalai Tesque), 150 μg/ml *D*-luciferin potassium salt (Cayman Chemical), 0.5 mM dithiothreitol (Nacalai Tesque), 50 μM ethylenediaminetetraacetic acid (pH8.0; Dojindo), and 0.1% Triton X-100 (Sigma-Aldrich). To calculate the inhibitory activities of colchicine and its derivatives on the ZFTA-RELA-responsive luciferase activity, the luciferase activity in 6E8 cells cultured in the presence or absence of doxycycline was designated as 0% or 100% inhibition, respectively. The IC_50_ values were calculated using the following formula:

IC_50_ = x2 × (x2/x1) ^((50 − y2)/(y2 − y1))^.

x1: sample concentration less than IC_50_.

x2: sample concentration higher than IC_50_.

y1: % inhibition at x1.

y2: % inhibition at x2.

### Quantitative PCR (qPCR)

cDNAs were prepared using NucleoSpin RNA (Macherey-Nagel) and ReverTra Ace (TOYOBO) kits, following the manufacturer’s instructions. Quantitative polymerase chain reaction (qPCR) was performed as previously described (14, 15, 24) using TB Green Premix Ex Taq II Tli RNaseH Plus (TaKaRa) on a StepOnePlus instrument (Thermo Fisher Scientific). The primers used for qPCR are listed in Table S3. TATA-binding protein (*TBP*) was used as an internal control.

### Immunocytochemistry

The 6E8 cells were seeded onto coverslips (Matsunami) coated with poly-*L*-lysine (Nacalai Tesque) and cultured overnight in culture media supplemented with 300 ng/ml doxycycline and the indicated compounds in 0.3% DMSO. Transfection was done using FuGENE6 transfection reagent (Promega) following the manufacturer’s instructions. Transfected 6E8 cells were treated with compounds at the indicated concentration 4 to 6 h following transfection and cultured overnight. To induce the nuclear localization of EGFP-RELA, 6E8 cells were treated with 10 ng/ml TNF-α (PeproTech) for 30 min before fixation. The cells were washed with Dulbecco’s phosphate-buffered saline (PBS) and fixed with 4% paraformaldehyde (PFA) dissolved in 100 mM phosphate buffer (PB, pH 7.4) at room temperature for 10 min. The fixed cells were washed with PBS and incubated in 10% normal goat serum (EMD Millipore) and 0.2% Triton X-100 (Sigma-Aldrich) in PBS (blocking buffer). The cells were incubated with rat anti-alpha tubulin (Novus Biologicals, Inc., clone YOL1/34), mouse anti-Flag (Merck, clone M2), rat anti-GFP (clone 6A11b) (58), or rat anti-HA (Merck, clone 3F10) antibodies diluted in blocking buffer at room temperature for 1 h, washed with PBS for 10 min three times, and incubated with either donkey anti-mouse or donkey anti-rat immunoglobulin G (IgG) conjugated with Alexa Fluor 488 (Abcam) and nuclear staining reagent [4′,6-diamidino-2-phenylindole (DAPI, Dojindo)] at room temperature for 1 h. The coverslips were washed in PBS at room temperature for 10 min three times and mounted with Aqua-Poly/Mount (Polysciences). Confocal microscopy images were acquired using a Nikon AX. Based on the morphological observations, the intracellular localization of the fusion proteins was evaluated in cells that were not undergoing cell death or mitosis. Nuclear localization ratios were calculated by dividing the degree of immunoreactivity observed in the nucleus by that observed in whole cells.

### Plasmid construction

pT2K ZFTA-MAML2/3-HA and ZFTA-NCOA2-HA were kind gifts from Dr Kristian Pajtler at the German Cancer Research Center and Dr Daisuke Kawauchi at Nagoya City University, respectively (9). pCGN-SRF (plasmid #11977, deposited by Dr Ron Prywes at Columbia University), p3XFLAG-MKL2 (#27175, Dr Ron Prywes), and GFP-ZMYND8 (#65401, Dr Kyle Miller at Emory University) were purchased from Addgene (59, 60, 61). The cDNAs were subcloned into the pCS2+ plasmid using the In-Fusion HD Cloning Kit (Clontech) based on the manufacturer’s instructions and fusion boundary sequences (12, 13, 37, 38).

### NLS prediction

To predict potential NLSs in ZFTA and RELA-fusion proteins, their entire regions were analyzed using cNLS Mapper (http://nls-mapper.iab.keio.ac.jp/) with a cutoff score of 4.0 (62).

### Statistics

Statistical analyses were done using Prism 9 (GraphPad) software. For luciferase-reporter, cell-viability, and qPCR assays, in which each data point represents an independent biological replicate, the means of three or more groups were compared using a one-way analysis of variance (ANOVA), followed by Tukey’s, Dunnett's, or uncorrected Fisher's LSD *post hoc* test as specified in each figure legend. For comparisons between two groups, an unpaired two-tailed Student’s *t* test was used. A *p*-value <0.05 was considered statistically significant.

For the immunofluorescence-based quantification of nuclear localization, data from two independent experiments are presented as SuperPlots (63): each small symbol represents an individual cell and each large symbol represents the mean of one independent experiment (biological replicate); the color scheme used to distinguish experiments and/or treatments is defined in each figure legend. The magnitude of each treatment effect is reported as the relative change in the nuclear localization ratio, calculated as the geometric mean of the per-experiment treatment-to-control ratios, and the direction of each effect was consistent across both independent experiments.

## Data availability

The data that support the findings of this study are available from the corresponding author upon reasonable request.

## Supporting information

This article contains supporting information.

## Declaration of generative AI and AI-Assisted technologies in the writing process

During the preparation of this work, the authors used DeepL and Claude in order to improve the readability of the manuscript. After using these tools, the authors reviewed and edited the content as needed and take full responsibility for the content of the publication.

## Conflict of interest

The authors declare that they have no conflicts of interest with the contents of this article.
