# Supplementary material for: Distinct nuclear localization signals confer differential microtubule dependence among ZFTA fusion oncoproteins
Source: J Biol Chem. 2026 Jul 13;302(9):113320. doi: 10.1016/j.jbc.2026.113320 (PMC13474238; doi:10.1016/j.jbc.2026.113320)
Supplement: Supplementary file 1 [file mmc2.pdf]

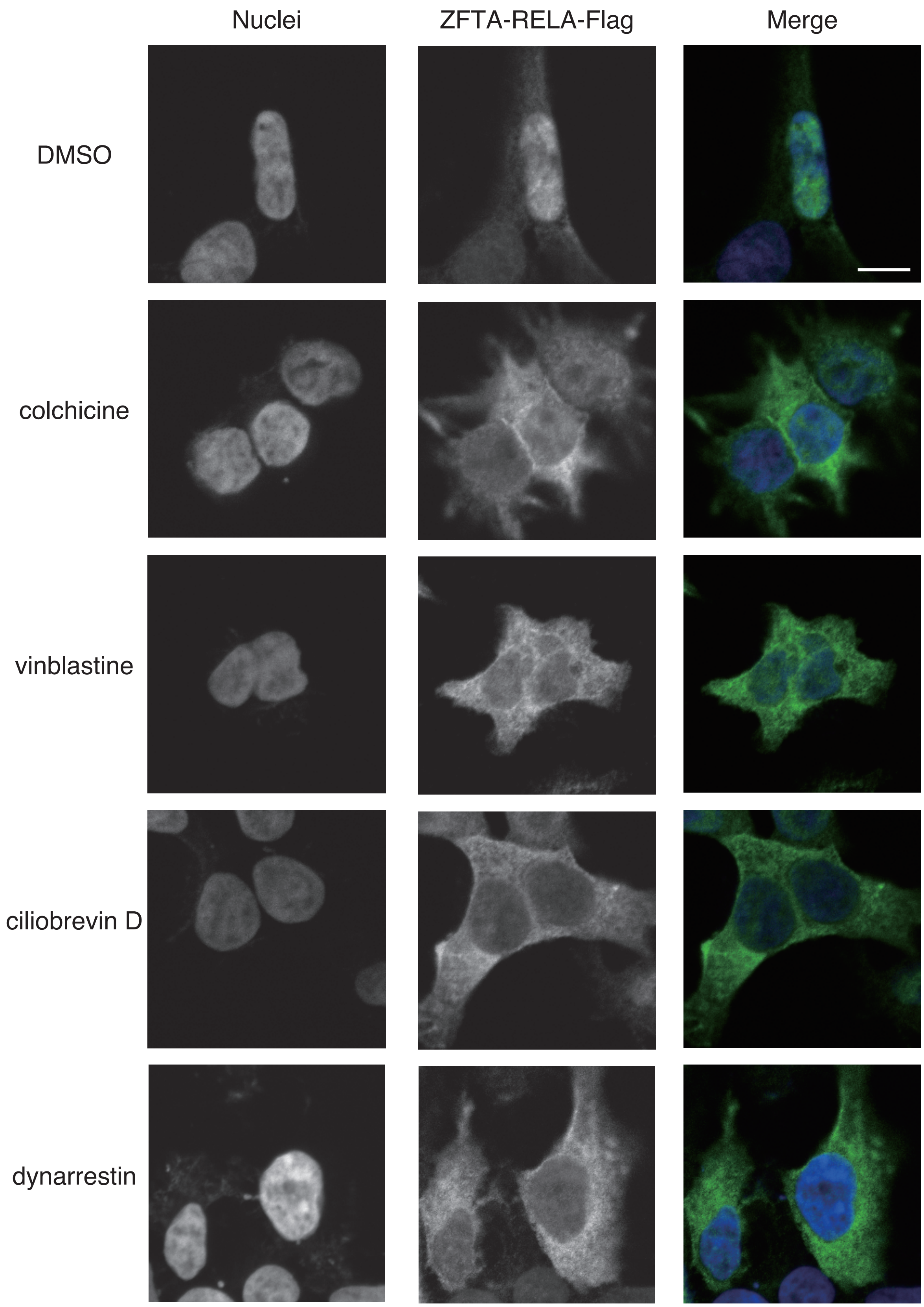
Nuclei
ZFTA-RELA-Flag
Merge
DMSO
colchicine
vinblastine
ciliobrevin D
dynarrestin

Figure S1

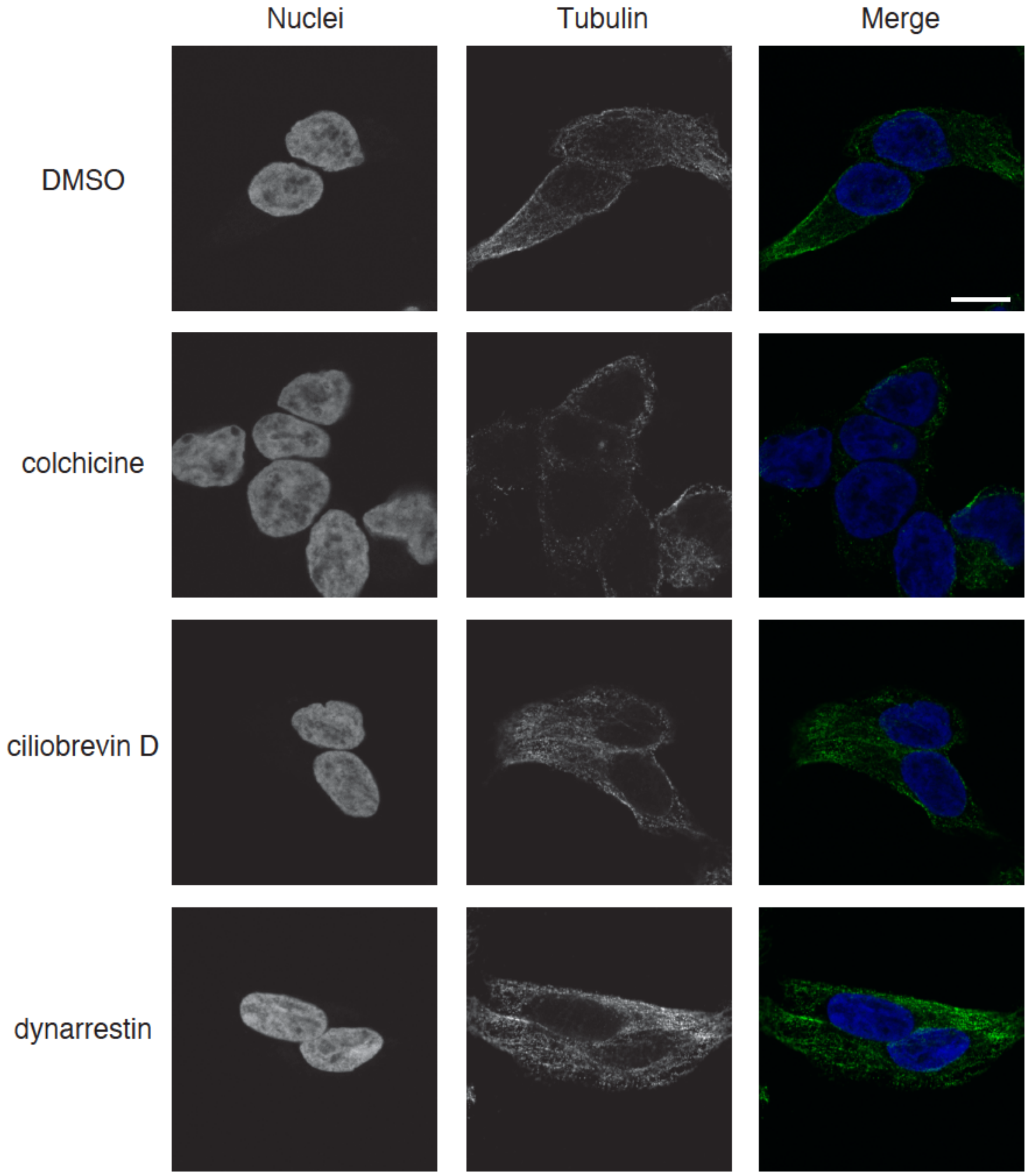
Nuclei
Tubulin
Merge
DMSO
colchicine
ciliobrevin D
dynarrestin

Figure S2

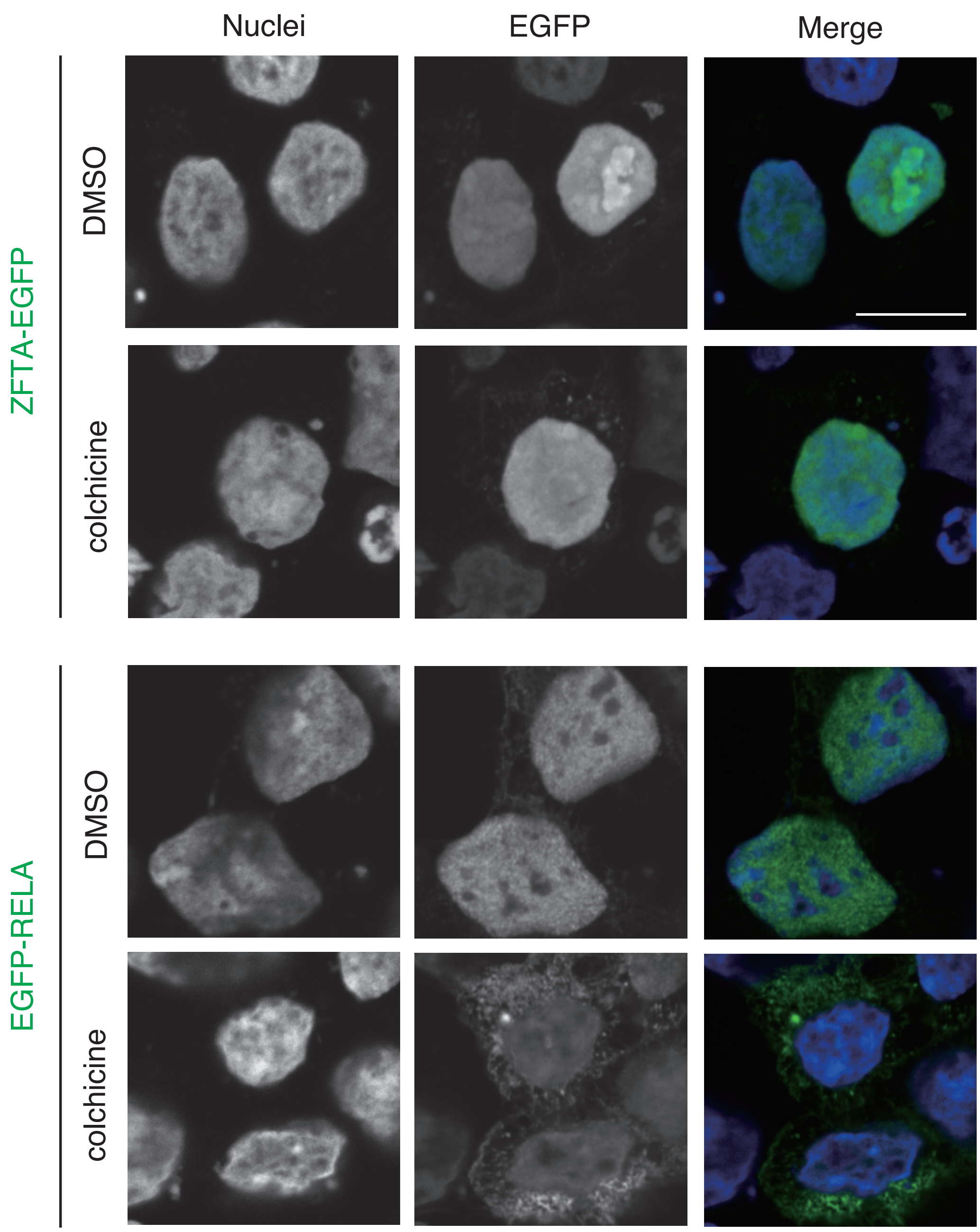
Nuclei
EGFP
Merge
ZFTA-EGFP
DMSO
colchicine
EGFP-RELA
DMSO
colchicine

Figure S3

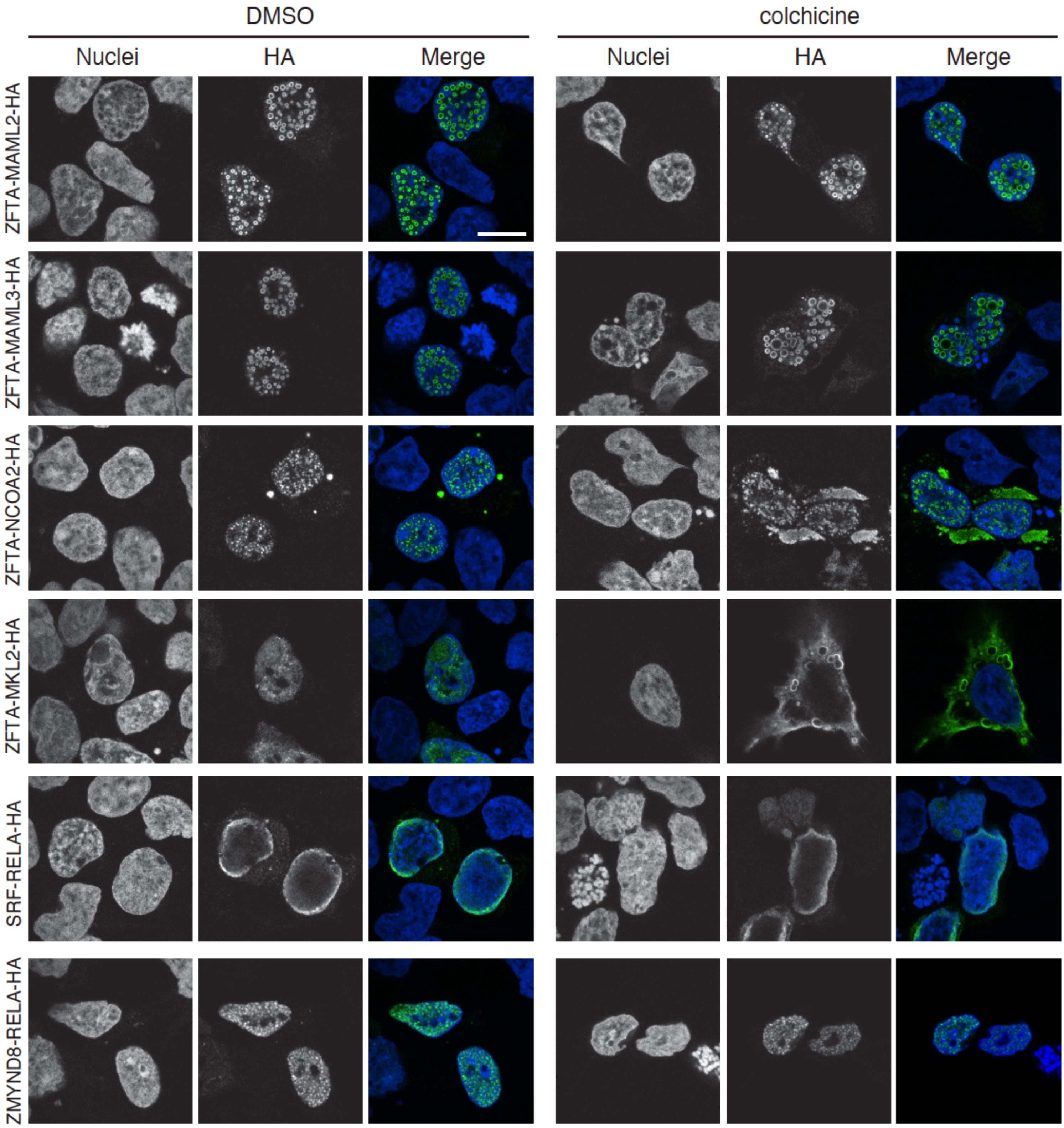
DMSO
colchicine
Nuclei
HA
Merge
Nuclei
HA
Merge
ZFTA-MAML2-HA
ZFTA-MAML3-HA
ZFTA-NCOA2-HA
ZFTA-MKL2-HA
SRF-RELA-HA
ZMYND8-RELA-HA

Figure S4

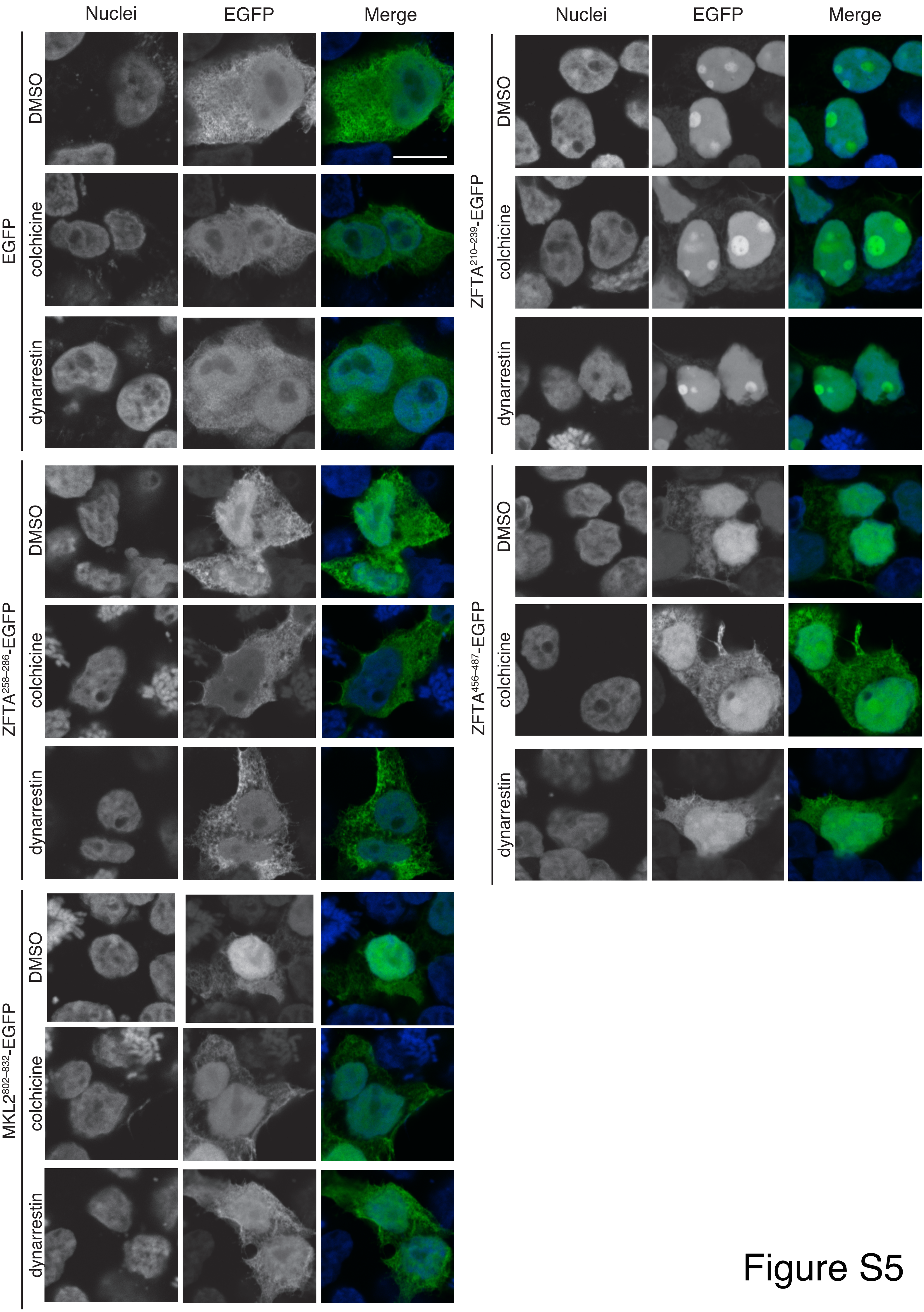
Nuclei
EGFP
Merge
EGFP
DMSO
colchicine
dynarrestin
ZFTA258–286-EGFP
MKL2802–832-EGFP
ZFTA210–239-EGFP
ZFTA456–487-EGFP

Figure S5
