# Supplementary material for: Distinct nuclear localization signals confer differential microtubule dependence among ZFTA fusion oncoproteins
Source: J Biol Chem. 2026 Jul 13;302(9):113320. doi: 10.1016/j.jbc.2026.113320 (PMC13474238; doi:10.1016/j.jbc.2026.113320)
Supplement: Supplementary file 2 [file mmc1.docx]

**Supporting Figure Legends**

**Figure S1. Single-channel grayscale images corresponding to Fig. 3A.**

Single-channel grayscale images and pseudo-colored merged images corresponding to Figure 3A are presented. The merged images are identical to those in Fig. 3A. Scale bar, 10 μm.

**Figure S2. Microtubule staining in 6E8 cells treated with dynein inhibitors.**

Immunofluorescence staining of α-tubulin (green) and nuclei (DAPI, blue) in 6E8 cells treated overnight with 0.3% DMSO (vehicle control), 0.10 µM colchicine, 30 µM ciliobrevin D, or 30 µM dynarrestin. Grayscale images of the nuclei and α-tubulin channel and the merged, pseudo-colored composite images (nuclei, blue; α-tubulin, green) are shown. Scale bar, 10 µm.

**Figure S3. Single-channel grayscale images corresponding to Fig. 4A.**

Single-channel grayscale images and pseudo-colored merged images corresponding to Figure 4A are presented. The merged images are identical to those in Fig. 4A. Scale bar, 10 μm.

**Figure S4. Single-channel grayscale images corresponding to Fig. 5B.**

Single-channel grayscale images and pseudo-colored merged images corresponding to Figure 5B are presented. Note that the merged images are identical to those in Fig. 5B. Scale bar, 10 μm.

**Figure S5. Single-channel grayscale images corresponding to Fig. 7B.**

Single-channel grayscale images and pseudo-colored merged images corresponding to Figure 7B are presented. Note that the merged images are identical to those in Fig. 7B. Scale bar, 10 μm.
