# Supplementary material for: Distinct nuclear localization signals confer differential microtubule dependence among ZFTA fusion oncoproteins
Source: J Biol Chem. 2026 Jul 13;302(9):113320. doi: 10.1016/j.jbc.2026.113320 (PMC13474238; doi:10.1016/j.jbc.2026.113320)
Supplement: Supplementary file 3 [file mmc3.docx]

Table S1. Inhibitory activities (%) of colchicine and its derivatives (1–6) on ZFTA-RELA-induced NF-κB-responsive luciferase activity.

| Inhibition rate (%) | | Concentration (µM) | | | | | |
| --- | --- | --- | --- | --- | --- | --- | --- |
|  |  | 0.032 | 0.10 | 0.32 | 1.0 | 3.2 | 10 |
| Compounds | Colchicine | -17 | 43 | 69 | 64 |  |  |
|  |  | 31 | 48 | 60 | 64 |  |  |
|  |  | 5 | 65 | 65 | 72 |  |  |
|  |  | 0.0 | 60 | 71 | 64 |  |  |
|  | 1 |  | 14 | 41 | 57 | 54 |  |
|  |  |  | 12 | 42 | 58 | 49 |  |
|  |  |  | 17 | 41 | 58 | 51 |  |
|  |  |  | 40 | 42 | 57 | 54 |  |
|  | 2 |  | 29 | 57 | 58 | 52 |  |
|  |  |  | 30 | 57 | 51 | 55 |  |
|  |  |  | 27 | 50 | 51 | 52 |  |
|  |  |  | 33 | 54 | 53 | 51 |  |
|  | 3 |  | 38 | 56 | 63 | 67 |  |
|  |  |  | 37 | 57 | 63 | 70 |  |
|  |  |  | 39 | 54 | 60 | 68 |  |
|  |  |  | 40 | 54 | 62 | 66 |  |
|  | 4 |  |  | -0.50 | 24 | 44 | 61 |
|  |  |  |  | 7.9 | 28 | 56 | 59 |
|  |  |  |  | 4.8 | 28 | 55 | 56 |
|  |  |  |  | 6.5 | 28 | 49 | 59 |
|  | 5 |  |  | -3.9 | 22 | 48 | 58 |
|  |  |  |  | 6.2 | 26 | 49 | 58 |
|  |  |  |  | -1.4 | 22 | 45 | 55 |
|  |  |  |  | 1.1 | 20 | 44 | 52 |
|  | 6 |  |  | 6.9 | 4.0 | 44 | 62 |
|  |  |  |  | 10.8 | -5.3 | 36 | 62 |
|  |  |  |  | 1.2 | -0.8 | 42 | 64 |
|  |  |  |  | -1.6 | -0.5 | 38 | 63 |

Table S2. Chemical compounds used in the present study

| Compounds | Supplier | Supplier ID | SMILES |
| --- | --- | --- | --- |
| Ciliobrevin D | Selleck | S9743 |  |
| Colchicine | FUJIFILM | C5645 |  |
| Dynarrestin | Cayman | 35527 |  |
| Vinblastine | Angene | AG019FX5 |  |
| **1** | ChemDiv | N051-0027 | COC1=CC=C2C(=CC1=O)[C@H](CCc3cc(OC)c(OC)c(OC)c23)N(C)CC#CCO |
| **2** | Alexis | 430-033 | CNC1CCc2cc(OC)c(OC)c(OC)c2C3=CC=C(OC)C(=O)C=C13 |
| **3** | ChemDiv | N051-0003 | COC1=CC=C2C(=CC1=O)[C@H](CCc3cc(OC)c(OC)c(OC)c23)N(C)Cc4ccccc4O |
| **4** | Pharmeks | P2000N-06164 | CN[C@H]1CCc2cc(OC)c(OC)c(OC)c2C3=CC=C(NCCO)C(=O)C=C13 |
| **5** | Pharmeks | P2000N-21042 | CN[C@H]1CCc2cc(OC)c(OC)c(OC)c2C3=CC=C(NCCCOC)C(=O)C=C13 |
| **6** | Pharmeks | P2000N-28889 | CNC1CCc2cc(OC)c(OC)c(OC)c2C3=C1C=C([O-])\C(=[N+](/C)\CC(OC)OC)\C=C3 |

Table S3. Primers used in the present study

| Primer Name | Sequence | PrimerBank ID |
| --- | --- | --- |
| *CCND1* F | 5′-CAATGACCCCGCACGATTTC-3′ | 77628152c3 |
| *CCND1* R | 5′-CATGGAGGGCGGATTGGAA-3′ |  |
| *CD82* F | 5′-GCTCATTCGAGACTACAACAGC-3′ | 67782353c2 |
| *CD82* R | 5′-GTGACCTCAGGGCGATTCA-3′ |  |
| *EPHB2* F | 5′-AGAAACGCTAATGGACTCCACT-3′ | 111118977c1 |
| *EPHB2* R | 5′-GTGCGGATCGTGTTCATGTT-3′ |  |
| *GLI2* F | 5′-CTGCCTCCGAGAAGCAAGAAG-3′ | 190343019c1 |
| *GLI2* R | 5′-GCATGGAATGGTGGCAAGAG-3′ |  |
| *L1CAM* F | 5′-TGTCATCACGGAACAGTCTCC-3′ | 221316755c1 |
| *L1CAM* R | 5′-CTGGCAAAGCAGCGGTAGAT-3′ |  |
| pCS2+ 3′UTR F | 5′-AGTCGTATTACGTAGATCCAGAC-3′ |  |
| pCS2+ 3′UTR R | 5′-CACAAATAAAGCATTTTTTTCACTGC-3′ |  |
| *PRKACA* F | 5′-AGCCCACTTGGATCAGTTTGA-3′ | 46909583c1 |
| *PRKACA* R | 5′-GTTCCCGGTCTCCTTGTGT-3′ |  |
| *TBP* F | 5′-GAGCCAAGAGTGAAGAACAGTC-3′ | 285026518c3 |
| *TBP* R | 5′-GCTCCCCACCATATTCTGAATCT-3′ |  |
| *ZFTA* F | 5′-GGGTCTGGAGGAAGAGATGCC-3′ |  |
| *ZFTA* R | 5′-TGCCCTCTGCTTTCCCACTCC-3′ |  |
